# Flesh Uniting

**Published:** 1867-12

**Authors:** 


### FLESH UNITING.

It is a common surgical operation to supply a new nose or ear or part of the face or lips from the arm, letting them grow together, then cut the arm loose, and manipulate the flesh into the proper shape.

An iron gate slammed too, while my neighbor's child was holding to the stationary upright; the mother saw it from her window, and running to her child she found the fleshy part of the end of the thumb cut off; with great presence of mind she pressed the severed pieces together; they united firmly. Our readers may make a practical use of these facts in many of the accidents of life; after all, these are the application of a principle of a common cut or gash, for if instantly the sides are pressed together and are kept together, the healing process goes on with great rapidity, leaving only a scar.

ILL SMELLING SORES, wounds, ulcers, cancer of the breast, etc. may be relieved of all bad smell by a painless dressing made of equal parts of Permanganate of Potash, powdered carbonate of lime and powdered starch.

HAIR REMOVED. Persons are sometimes annoyed by hairs growing in unsightly places; may be removed. Thus, take sixty grains of quick lime, forty grains of yellow sulphate of arsenic and sixty grains of powdered starch, make it into a paste with water, apply it to the spot from which it is desired to remove he hair, and in a few minutes it is done.

